# The chondroitin sulfate moiety mediates thrombomodulin-enhanced adhesion and migration of vascular smooth muscle cells

**DOI:** 10.1186/s12929-018-0415-7

**Published:** 2018-02-13

**Authors:** Vincent Chunpeng Pai, I-Chung Lo, Yan wun Huang, I-Ching Tsai, Hui-Pin Cheng, Guey-Yueh Shi, Hua-Lin Wu, Meei Jyh Jiang

**Affiliations:** 1 Department of Cell Biology and Anatomy, College of Medicine, National Cheng Kung University, 1 Ta-Hsueh Road, Tainan, 70101 Taiwan; 2 Cardiovascular Research Center, College of Medicine, National Cheng Kung University, Tainan, 70101 Taiwan; 3 Department of Biochemistry and Molecular Biology, College of Medicine, National Cheng Kung University, Tainan, 70101 Taiwan

**Keywords:** Thrombomodulin, Chondroitin sulfate moiety, Migration, Adhesion, Vascular smooth muscle cells

## Abstract

**Background:**

Thrombomodulin (TM), a transmembrane glycoprotein highly expressed in endothelial cells (ECs), is a potent anticoagulant maintaining circulation homeostasis. Under inflammatory states, TM expression is drastically reduced in ECs while vascular smooth muscle cells (VSMCs) show a robust expression of TM. The functional role of TM in VSMCs remains elusive.

**Methods:**

We examined the role of TM in VSMCs activities in human aortic VSMCs stimulated with platelet-derived growth factor-BB (PDGF-BB). Using rat embryonic aorta-derived A7r5 VSMCs which do not express TM, the role of the chondroitin sulfate (CS) moiety of TM in VSMCs was delineated with cells expressing wild-type TM and the CS-devoid TM mutant.

**Results:**

Expression of TM enhanced cell migration and adhesion/spreading onto type I collagen, but had no effect on cell proliferation. Knocking down TM with short hairpin RNA reduced PDGF-stimulated adhesion and migration of human aortic VSMCs. In A7r5 cells, TM-mediated cell adhesion was eradicated by pretreatment with chondroitinase ABC which degrades CS moiety. Furthermore, the TM mutant (TM^S490, 492A^) devoid of CS moiety failed to increase cell adhesion, spreading or migration. Wild-type TM, but not TM^S490, 492A^, increased focal adhesion kinase (FAK) activation during cell adhesion, and TM-enhanced cell migration was abolished by a function-blocking anti-integrin β_1_ antibody.

**Conclusion:**

Chondroitin sulfate modification is required for TM-mediated activation of β_1_-integrin and FAK, thereby enhancing adhesion and migration activity of VSMCs.

**Electronic supplementary material:**

The online version of this article (10.1186/s12929-018-0415-7) contains supplementary material, which is available to authorized users.

## Background

Migration and proliferation of vascular smooth muscle cells (VSMCs) are key events in the intimal thickening of atherosclerosis and neointimal formation induced by vascular injury [1]. During vascular remodeling, VSMCs in the media transform from a quiescent, contractile phenotype into an activated, synthetic phenotype and migrate to the intima. VSMCs in the intima may undergo replication, active production of extracellular matrix (ECM) or even secretion of pro-inflammatory factors, resulting in intimal thickening and atherosclerosis progression. Aberrant migration and proliferation of VSMCs can be triggered by excessive secretion of various cytokines and growth factors [2].

Thrombomodulin (TM), a transmembrane glycoprotein in endothelial cells (ECs), is a critical player in maintaining vascular thromboresistance. TM acts as a cofactor in protein C activation through regulating thrombin substrate affinity and plays a critical role in the anticoagulant pathway [3]. Under physiological conditions, TM is highly expressed on the endothelial surface and is considered an endothelial marker. In disease states associated with inflammation, such as atherosclerosis and vein graft thrombosis, TM expression in ECs is markedly downregulated, leading to thrombotic complications and an increase in local inflammation and matrix proteolysis [4, 5]. Interestingly, in advanced atherosclerotic plaques, TM is detected in VSMCs of both the intima and the media [6, 7]. Thus, intimal VSMCs were proposed to be a relevant source of TM and form a surface relatively resistant to thrombosis. We previously found that platelet-derived growth factor (PDGF)-BB, the most potent mitogen and chemoattractant of VSMCs in vitro, induces TM expression in VSMCs through Src kinase and PI3-kinase/Akt/mTOR-dependent signaling pathway. Furthermore, PDGF-BB and TM co-localize in the media and the neointima following carotid ligation-induced injury [8]. Similarly, TM expression is induced in distension-caused urinary bladder injuries and is involved in the PDGF-stimulated migration of urinary bladder smooth muscle cells [9]. Recent evidence further showed that the forced expression of TM in TM-deficient A2058 melanoma cells enhances cell adhesion, migration, and invasion through the activation of focal adhesion kinase (FAK) [10].

TM consists of five domains, including an N-terminal lectin-like domain, followed by six epidermal growth factor (EGF)-like repeats, a serine/threonine–rich domain, a transmembrane domain, and a cytoplasmic domain [11]. The activation of protein C requires thrombin binding to the EGF-like domain and is enhanced by a second site binding to the chondroitin sulfate (CS) moiety attached to the serine/threonine–rich domain [12]. In addition to an anticoagulant function, previous studies have revealed multiple biological roles for TM including anti-inflammation [13], cell-cell adhesion [14], cell-matrix adhesion and migration [10], cell proliferation, and angiogenesis [15], which are linked to different domains of TM. While TM peptides consisting of EGF-like repeats and serine/threonine-rich domains stimulate cell proliferation, migration, and angiogenesis [15], whether the CS moiety of TM participates in these processes remains unclear. Previous studies have suggested that proteoglycans containing CS play a significant role in regulating cell adhesion and migration in a variety of cell types [16–19]. The CS moiety on CD44, a transmembrane glycoprotein, modulates cell migration on type I collagen in melanoma cells, which is abolished by chondroitinase ABC (ChABC), an enzyme that cleaves CS from the core protein [16]. Similarly, CD44-associated CS proteoglycan mediates EC migration and adhesion on fibrinogen [17]. In addition, CS proteoglycans regulate cell growth, differentiation, and migration of neural precursor cells [20].

We hypothesized that the CS moiety of TM has functional roles in VSMCs adhesion and migration analogous to the CS of other membrane proteins. We tested our hypothesis by examining the effect of ChABC and the CS-devoid TM mutant expression on VSMCs adhesion and migration. Our results indicate that TM expression promotes cell spreading, adhesion, and migration involving the activation of β_1_-integrin and FAK and the CS moiety is required for these processes.

## Methods

### Cell culture

Human aortic VSMCs (HASMCs) purchased from Cascade Biologics (Portland, OR, USA) were amplified in medium 231 with smooth muscle growth supplement (Cascade Biologics). A7r5 cells were purchased from the American Type Culture Collection (Manassas, VA, USA). HASMCs and A7r5 cells were cultured in Dulbecco’s modified Eagle’s medium (DMEM, Life Technologies, Inc.) containing 10% fetal bovine serum (FBS, HyClone), 25 mM HEPES, Glutamax, 100 U/ml penicillin, and 100 μg/ml streptomycin at 37 °C under 5% CO_2_ humidified atmosphere. HASMCs were used between 3 and 6 passages.

### Lentivirus-based knockdown of TM expression

pLKO.1-puro-shTM, pCMV-ΔR8.91 and pMD.G were obtained from National RNAi core facility (Academia Sinica, Taiwan). The lentivirus stock was prepared as previously described [21]. HASMCs were infected with a viral stock in complete medium for 5 days and were processed for the subsequent experiments.

### Transient transfection

A7r5 cells at 40-60% confluence were transfected with Fugene HD (Roche Applied Science) at a 2.5:1 ratio to plasmid DNA. The amount of DNA was adjusted if necessary. The medium was replaced 12 or 24 h later, and the cells were harvested 48 or 72 h later. Transfection efficiency was consistently 30–50% as determined by green fluorescent protein expression.

### Immunoblotting analysis

Following treatments, VSMCs were lysed and immunoblotting was performed as previously described [22]. TM, FAK phosphorylation (Tyr397), and FAK expression were detected by incubation with a primary antibody for TM (mouse clone D-3, Santa Cruz Biotechnology), pFAK-Y397 (rabbit polyclonal antiserum, Upstate) or FAK (rabbit polyclonal antiserum, BD Transduction Lab.), followed by horseradish peroxidase (HRP)-conjugated goat anti-mouse or anti-rabbit IgG (Vector Laboratories). Protein bands were visualized by enhanced chemiluminescence using ECL-Plus (PerkinElmer, Inc.) and quantified densitometrically. Expression of α-tubulin (mouse clone B-5-1-2, Sigma) or β-actin (mouse clone AC-15, Sigma) was used as loading control.

### Site-directed mutagenesis

TM DNA within the pEGFP-N1 vector was mutated at serine 490 and 492 to alanine using the QuikChange™ site-directed mutagenesis kit from Stratagene (La Jolla, CA, USA) following the manufacturer’s instructions. DNA sequencing confirmed the mutations.

### Adhesion assay

The ability of VSMCs to adhere to ECM molecules was quantified as previously described [23]. Clear-bottom 96-well plates were coated with 40 μg/ml collagen type I or 5 μg/ml fibronectin in PBS for 16 h at 4 °C. Non-specific binding was blocked with 2% BSA in PBS. HASMCs were stimulated with 10 ng/ml human recombinant PDGF-BB (R&D Systems) for 6 h before adhesion assay. A7r5 cells were transfected with 1 μg of pEGFP, pEGFP-TM, or pEGFP-TM^S490,492A^ for 12-24 h and cultured in regular medium for 36-48 h. Cells were trypsinized, washed, and treated both with and without ChABC (0.5 U/ml) for 1 h at 37 °C. The cells (10^4^ cells/100 μl) were added to the coated 96-well plates and incubated for 30 min at 37 °C. Non-adherent cells were removed by washing with DMEM. Adherent cells were fixed with 4% paraformaldehyde, stained with 0.1% crystal violet for 25 min, and solubilized with 0.5% triton X-100. The optical density at 595 nm (OD 595) was determined using a microplate reader.

### Wound healing assay

Fully confluent cell cultures were wounded with a 100-μl sterile pipette tip as previously described [24]. Wound healing assay was conducted in a CO_2_ incubator. Each scratch was randomly photographed at four separate sites along the length with time-lapse video microscopy over the succeeding 24 h. Migration velocity of A7r5 cells expressing GFP, TM-GFP, or TM^S490, 492A^-GFP across the wound edges was measured manually. In some experiments, a hamster IgM (clone Ha2/5, BD Pharmingen) or the control IgM (G235-1) was used. The migration rate of HASMCs was determined by the ratio of the wound area at 24 h to the initial wound area.

### Invasion assay

To determine the invasive ability of A7r5 cells, the upper sides of the transwell polycarbonate membrane filters, with 8-μm pore size (Corning Inc.), were coated with 40 μg/ml collagen type I or 5 μg/ml fibronectin. Cells (20,000/well) in serum-free medium were seeded in the upper chamber, and the bottom chamber contained medium with 10% FBS. Cells were incubated for 16 h at 37 °C. Following incubation, the cells that had invaded and attached to the lower surface of the membrane were fixed with 4% paraformaldehyde and stained with 4′,6-diamidino-2-phenylindole (DAPI, diluted 1000× in PBS) (Sigma-Aldrich). Cell number was counted in five random fields per membrane under an inverted microscope.

### TM and pFAK immunofluorescence

HASMCs were serum-starved for 36 h and treated with or without PDGF-BB (10 ng/ml) for 6 h. A7r5 cells were transfected with pEGFP, pEGFP-TM or pEGFP-TM^S490,492A^ (1 μg) for 12 h and cultured in fresh medium for 36-60 h. Cells were detached and seeded onto 40 μg/ml type I collagen-coated cover slip for 3 h. The cells were fixed for 10 min with 4% paraformaldehyde and permeabilized for 15 min with 0.1% Triton X-100 in phosphate buffered saline (PBS)/BSA. The cells were incubated at 4 °C overnight with anti-TM (mouse clone D-3, Santa Cruz Biotechnology) or anti-pFAK^Y397^ antibody (BD611807, 1:100 in 3% BSA/PBS), followed by Alexa 546–conjugated goat anti–mouse IgG (Molecular Probes; 1:200). The cell nuclei were stained with DAPI, and cells were observed under a confocal microscope (Leica *TCS SP5* II).

### Ki67 immunofluorescence staining

A7r5 cells were transfected with pEGFP, pEGFP-TM or pEGFP-TM^S490,492A^ for 12 h and cultured for 12 h. Following a 48 h serum starvation, cells were treated with 10 ng/ml PDGF-BB for 24 h. The cells were fixed for 10 min with 4% paraformaldehyde and permeabilized for 15 min with 0.1% Triton X-100 in PBS/BSA. The cells were incubated for 1 h with an anti-Ki-67 antibody (Novocastra, NCL-Ki67-MM1, 1:100 in 3% BSA/PBS), followed by Alexa 546–conjugated goat anti–mouse IgG (Molecular Probes; 1:100). The nuclei were stained with DAPI, and cells were observed under an inverted fluorescence microscope (Leica IRE-2).

### Construction of lentivirus-based GFP-tagged TM and TM^S490, 492A^

Human TM and TM^S490, 492A^ were amplified and subcloned from pEGFP-N1-TM vector [14] and pEGFP-N1-TM^S490, 492A^ vector. TM-EGFP and TM^S490, 492A^-EGFP fragments were cut from pEGFP-N1 using EcoRI restriction endonuclease. pLVX-TM-GFP-puro and pLVX-TM^S490, 492A^-GFP-puro vectors were generated by subcloning the TM-EGFP and TM^S490, 492A^-EGFP into pLVX-IRES-puro (Clontech) vector pre-treated with EcoRI. Sequences of both constructs were confirmed by DNA sequencing.

### Establishing stable cell lines

For lentivirus production, plasmids pXPAS2, pMD2G, and pLVX-TM-GFP-puro (or pLVX-TM^S490, 492A^-GFP-puro) were co-transfected into 293 T cell with Fugene HD, and supernatants containing lentiviral particles were collected at 48, 72, and 96 h following transfection. A7r5 cells at 50-60% confluence were transduced with lentivirus-containing supernatants. At 48 h post-transduction, 1 μg/ml puromycin was added to select cells stably expressing TM-GFP, TM^S490, 492A^-GFP or vehicle.

### Statistical analysis

Data are presented as mean ± SEM of n independent experiments. Statistical analysis was performed with Student’s *t* test for comparison between two groups. For comparisons among multiple groups, one-way ANOVA, followed by Dunnett multiple comparison was used. *P* values smaller than 0.05 were considered significant.

## Results

### VSMCs expressed TM both with and without chondroitin sulfate (CS) moiety

**Fig. 1:**
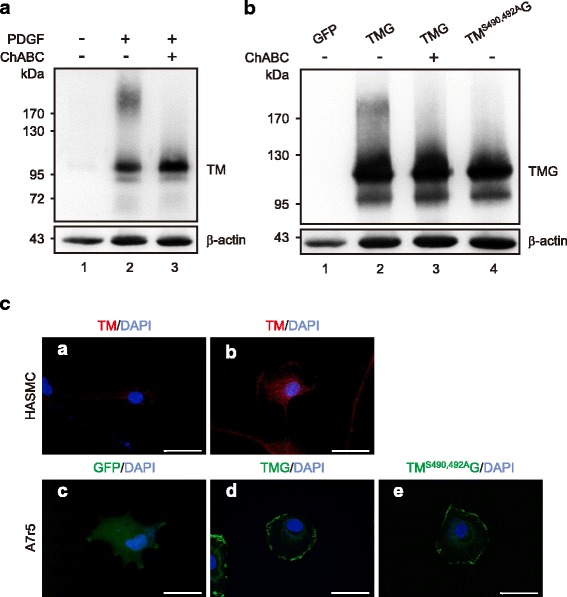
Thrombomodulin (TM) expression, glycosylation, and localization in HASMCs and A7r5 transfected with TM cDNA constructs. **a** HASMCs were serum-starved for 48 h (Lane 1) and then stimulated with PDGF-BB (10 ng/ml) for 6 h without (Lane 2) or with (Lane 3) ChABC (0.5 U/ml) treatment for 1 h. **b** A7r5 cells were transfected with pEGFP (Lane 1), pEGFP-TM (Lane 2) or pEGFP-TM^S490,492A^ (Lane 4). The cells overexpressing TM were treated with ChABC before harvest (Lane 3). Western blotting was performed with antibodies specific to either human TM (Top) or β-actin (Bottom). **c** The localization of TM in VSMCs. **a** and **b**, HASMCs were serum-starved for 36 h and treated without (**a**) or with (**b**) PDGF-BB for 6 h. **c, d,** and **e**, A7r5 cells transfected with pEGFP (**c**), pEGFP-TM (**d**) or pEGFP- TM^S490,492A^ (**e**) were fixed, processed for TM immunofluorescence (**a** and **b**), and examined under a confocal microscope. Scale bar: 50 μm

We previously reported that HASMCs express TM under PDGF stimulation but not at quiescence [8]. On the contrary, A7r5 cells did not express TM mRNA in the presence or absence of PDGF treatment (Additional file 1: Supplementary data, Table S1 and Figure S1). Therefore, we applied both HASMCs and A7r5 cells to examine the functional roles of TM in VSMCs. We first examined TM expression in PDGF-stimulated HASMCs and A7r5 cells transfected with TM cDNA. Compared to quiescent cells (Fig. 1a, Lane 1, serum starvation for 48 h), PDGF treatment profoundly increased TM expression in HASMCs. TM mainly existed as a ~ 100 kDa form, but a diffused, high-molecular-mass band of approximately 180-200 kDa was also present (Fig. 1a, Lane 2). TM possesses four potential sites for O-linked glycosylation, which supports the post-translational attachment of a CS moiety, a stretch of approximately 20 repeating disaccharide units with a trisaccharide terminus [25]. HASMCs treated with ChABC (0.5 U/ml) substantially reduced the high-molecular-mass form (Fig. 1a, Lane 3), indicating that TM expressed in HASMCs was modified by CS.

The CS moiety of TM is indispensable for TM to perform its anticoagulant activity [12]. However, additional roles for the CS moiety of TM have not been defined. To address this question, we generated the TM mutation of Ser^490^ and Ser^492^ to Ala, which was shown to block the CS modification of TM [26]. Transfection with pEGFP-TM^S490, 492A^ had no effect on TM expression (Fig. 1b, Lanes 2 and 4). A7r5 cells expressing TM-GFP exhibited a pattern similar to that of PDGF-stimulated HASMCs with both bands showing higher molecular mass resulting from the GFP tag. The high-molecular-mass bands were eliminated with ChABC treatment. In addition, A7r5 cells expressing TM^S490, 492A^ exhibited only the low-molecular-mass band. These results suggested that TM underwent CS modification in both HASMCs and A7r5 cells through serine 490 and 492. The cellular localization of TM in PDGF-stimulated HASMCs and TM-expressing A7r5 cells was examined. TM was mainly localized on the plasma membrane (Fig. 1c, b & d) and CS-deficient TM^S490, 492A^ displayed a similar distribution to wild-type TM (Fig. 1c, e).

### The CS moiety mediates TM-enhanced VSMCs adhesion and spreading

**Fig. 2:**
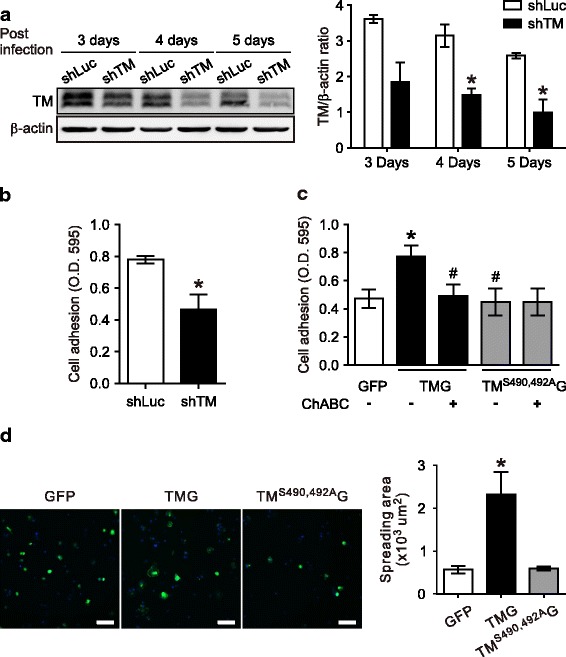
Thrombomodulin enhanced VSMCs adhesion and spreading to type I collagen in a chondroitin sulfate-dependent manner. **a** HASMCs were infected with lentiviral shRNA constructs targeting luciferase (shLuc) or TM (shTM) for 1-3 days, serum-starved for 2 days, and treated with PDGF for 6 h. TM expression levels were analyzed with Western blotting using β-actin as the loading control. The top panel shows a representative result and the bottom panel summarizes the results of three independent experiments expressed as mean ± SEM. **b** HASMCs infected with lentivirus carrying luciferase or TM shRNA were seeded on a 96-well plate coated with type I collagen, allowed to adhere for 0.5 h at 37 °C, and stained with crystal violet. Results were obtained from 4 independent experiments and expressed as mean ± SEM, *P < 0.05 compared with shLuc. **c** and **d** A7r5 cells transfected with pEGFP, pEGFP-TM, or pEGFP-TM^S490,492A^ were cultured for 36 h. In (**c)**, cells were treated with or without ChABC (0.5 U/ml) for 1 h before adhesion assay. Results were obtained from six independent experiments and expressed as mean ± SEM. In (**d)**, cells were detached, seeded to type I collagen-coated dish for 30 min, and processed for fluorescence microscopy. The left panel shows representative results for cells expressing GFP, TM-GFP, and TM^S490,492A^-GFP (green) with DAPI nuclear staining; the right panel summarizes the spreading area for each group from at least 3 independent experiments. *P < 0.05, compared with the GFP-expressing group; #*P* < 0.05, compared with the TM-GFP-expressing group. Scale bar: 100 μm

TM functions as a Ca^2+^-dependent cell-to-cell adhesion molecule [14] and was recently found to bind to fibronectin through the lectin-like domain [10]. To assess the effect of TM on VSMCs adhesion to the ECM, we performed cell adhesion assay on plates coated with type I collagen which is a major ECM in the neointima [27]. HASMCs were infected with a lentiviral vector encoding luciferase shRNA (shLuc) or TM shRNA (shTM). The infection with shTM, but not shLuc, for 3 to 5 days markedly decreased TM expression in HASMCs, showing the specificity of the engineered lentivirus (Fig. 2a). The adhesion of TM-depleted HASMCs was markedly suppressed (41.0% ± 9.6% of control, *P* < 0.05) (Fig. 2b). Cell surface CD44-related CS proteoglycan was reported to mediate melanoma cell adhesion and migration on type I collagen [16]. Therefore, we hypothesized that TM affects the adhesive and/or migratory capabilities of VSMCs through the CS component and examined it with A7r5 adhesion onto type I collagen. Expression of TM-GFP significantly increased A7r5 adhesion (Fig. 2c, 163.5% ± 15.9% of GFP, *P* < 0.01), which was reversed by ChABC treatment (26.3% ± 5.7% of TM-GFP, *P* < 0.05). Moreover, expression of TM^S490, 492A^-GFP had no effect on cell adhesion onto type I collagen. To examine whether TM-enhanced cell adhesion of VSMCs is specific to type I collagen, we compared A7r5 cells adhesion to type I collagen-, fibronectin-, and non-coated surface. TM expression markedly enhanced cell adhesion to both ECM molecules with stronger effect detected when fibronectin was used. In contrast, expression of TM^S490, 492A^-GFP inhibited cell adhesion onto fibronectin. As a comparison, expression of another TM mutant with lectin-like domain deletion (TMΔL-GFP) enhanced cell adhesion to both molecules less effectively compared to TM-GFP expression (Additional file 1: Supplementary data, Figure S2). These results indicated that TM promotes VSMCs adhesion and the CS moiety mediates the effect. Next, we examined whether TM affects cell spreading. A7r5 cells expressing GFP, TM-GFP or TM^S490, 492A^-GFP were re-suspended and allowed to adhere onto immobilized type I collagen for 30 min. Cells expressing TM-GFP exhibited accelerated spreading and appeared flattened with extended lamellipodia. In contrast, most GFP- and TM^S490, 492A^-GFP-expressing cells remained round. The spreading area of TM-expressing cells was increased (2.3 ± 0.18 fold control), whereas cells expressing CS-deficient TM^S490, 492A^ showed no difference in spreading area (Fig. 2d).

### The CS moiety is required for TM-enhanced VSMCs migration

**Fig. 3:**
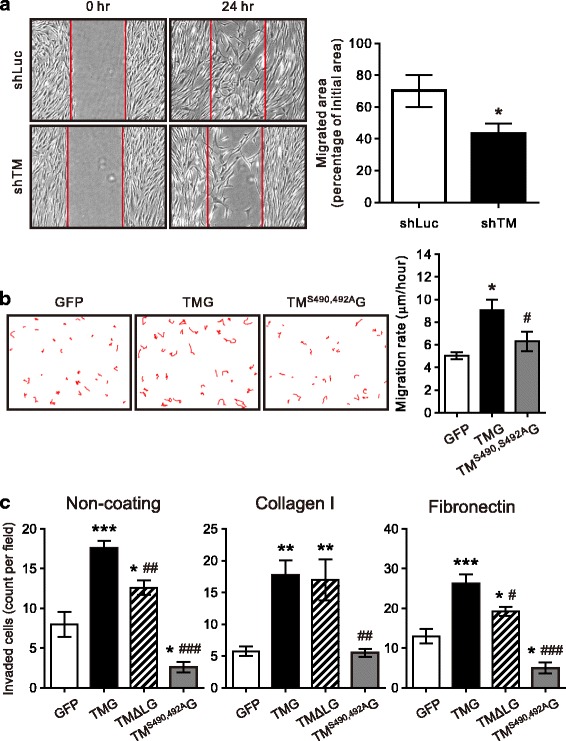
Thrombomodulin is involved in VSMCs migration. **a** HASMCs infected with a lentivirus encoding luciferase (shLuc) or TM (shTM) shRNA were cultured in regular medium. Cell migration was analyzed by a wound-healing assay. Each monolayer was scratched, incubated for 24 h, and followed by image acquisition. The open area (scratch) was quantified with ImageJ software from 5 different experiments and expressed as mean ± SEM. *P < 0.05 compared with shLuc. **b** A7r5 cells transfected with pEGFP, pEGFP-TM or pEGFP-TM^S490, 492A^ were cultured for 36 h and serum-starved for 24 h. Wound healing assay was performed under PDGF-BB treatment for 24 h. Cell motility was quantified from 4 independent experiments and expressed as mean ± SEM. **c** A7r5 cells transfected with pEGFP, pEGFP-TM, pEGFP-TMΔL or pEGFP-TM^S490, 492A^ underwent invasion assay in transwells without coating or coated with type I collagen or fibronectin. Cells seeded in the upper chamber were incubated for 16 h, those attached to the lower surface of the membrane were fixed, stained with DAPI, and GFP-expressing cells counted. *P < 0.05, **P < 0.01, ***P < 0.001, compared with the GFP-expressing group; ^#^*P* < 0.05, ^##^*P* < 0.01, ^###^*P* < 0.001, compared with the TM-GFP-expressing group

VSMCs migration across the internal elastic lamella into the intima is a key process in neointima formation [28]. To investigate the role of TM in VSMCs migration, wound healing assay was performed under PDGF treatment for 24 h in HASMCs infected with lentivirus encoding luciferase or TM shRNA. Knocking down TM expression in HASMCs attenuated wound closure compared to the control group (Fig. 3a, TM knockdown: 40.4% ± 6.1%; Luc knockdown: 70.5% ± 9.7%, *P* < 0.05). To further elucidate the role of the TM CS moiety in VSMCs migration, A7r5 cells expressing TM-GFP, TM^S490, 492A^-GFP or GFP were analyzed with wound-healing assay. Cells expressing TM-GFP exhibited markedly higher migration velocity (8.6 ± 0.72 μm/h) compared with the GFP control (5.0 ± 0.3 μm/h), whereas cells expressing TM^S490, 492A^-GFP (6.3 ± 0.9 μm/h) showed no effect on cell mobility (Fig. 3b). We further examined the effect of expressing TM-GFP, TM^S490, 492A^-GFP, and TMΔL-GFP on A7r5 cells invasion through type I collagen- and fibronectin-coated transwells. Similar to that found with adhesion assay, TM-enhanced cell invasion was more pronounced with fibronectin coating. Expression of TM^S490, 492A^-GFP inhibited cell invasion through fibronectin-coated wells, whereas TMΔL-GFP was less effective than TM-GFP in enhancing cell invasion (Fig. 3c). These results indicated that the CS moiety mediates TM-promoted mobility of VSMCs.

### TM-enhanced VSMCs migration involves β_1_-integrin and focal adhesion kinase

**Fig. 4:**
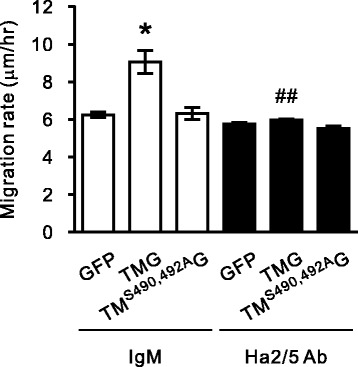
The blockade of β1-integrin function eliminated thrombomodulin-enhanced VSMCs migration. A7r5 cells transfected with pEGFP, pEGFP-TM or pEGFP-TM^S490, 492A^ were cultured for 36 h and serum-starved for 24 h. A wound healing assay was performed under PDGF treatment for 24 h after pretreating cells with 20 mg/mL of control IgM or anti-β1 integrin antibody, Ha2/5. Cell motility was quantified from at least 3 different experiments and expressed as mean ± SEM. *P < 0.05, compared with the GFP-expressing group; ^##^*P* < 0.01, compared with the TM-GFP-expressing group

CS on CD44 is implicated in mediating EC adhesion and migration [17]. Furthermore, another CS-containing glycoprotein, PG-M/versican, binds to β_1_-integrin, thereby activating FAK and cell adhesion [29]. We next explored whether β_1_-integrin is involved in TM-increased cell mobility. A7r5 cells expressing GFP-tagged TM or TM^S490, 492A^ were applied to wound healing assay in the presence of PDGF and control IgM or a function-blocking antibody against rat integrin β_1_ (Ha 2/5). Blocking integrin β_1_ activation eliminated the migration promoted by TM-GFP, whereas the mobility of cells expressing TM^S490, 492A^-GFP was not affected (Fig. 4). These results suggest that TM-promoted cell migration is mediated by β_1_ integrin activation and CS modification is required for this effect.

**Fig. 5:**
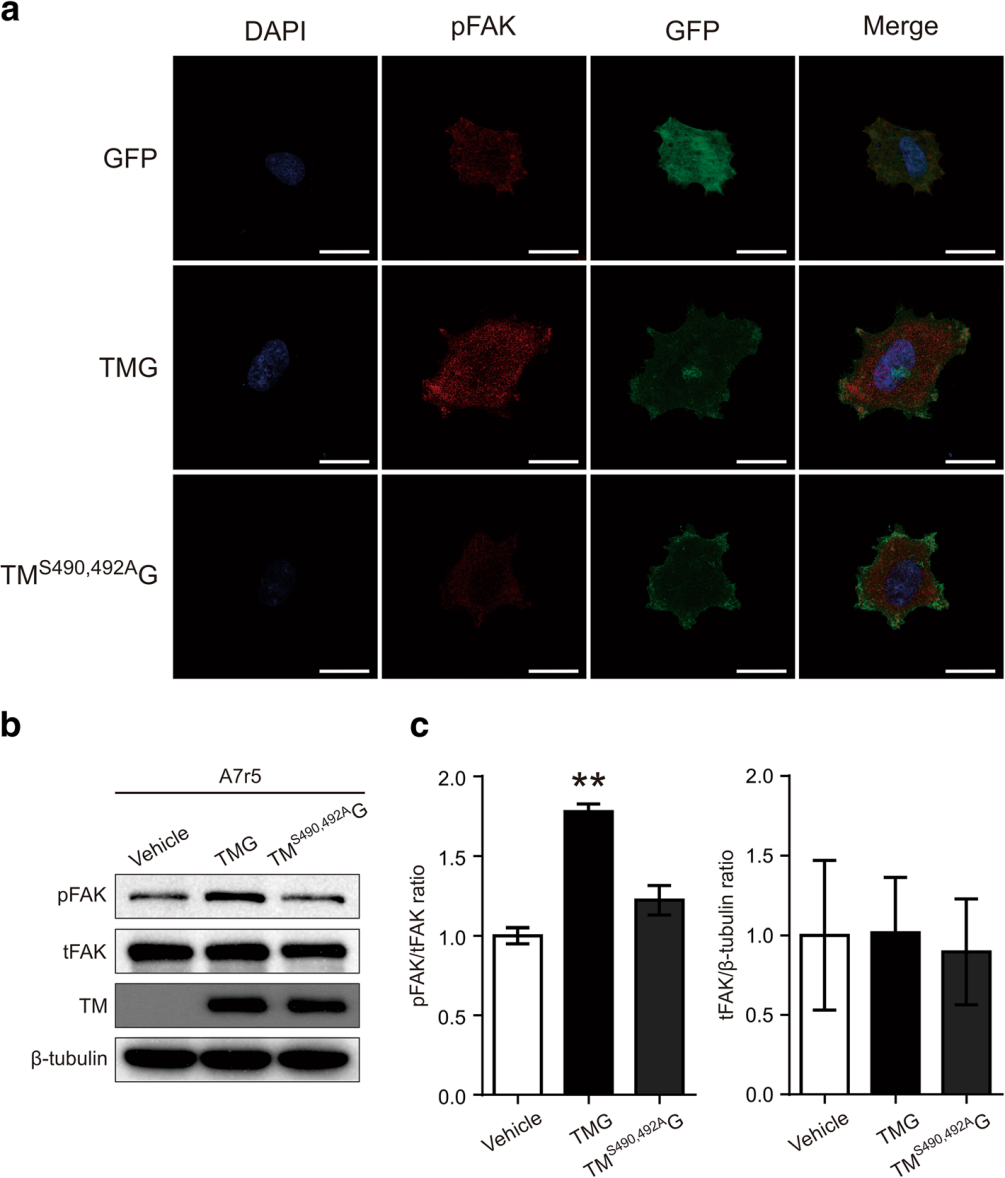
Thrombomodulin (TM) expression increased FAK phosphorylation in A7r5 cells upon adhesion. **a** A7r5 cells transfected with pEGFP, pEGFP-TM, or pEGFP-TM^S490,492A^ were detached and seeded to type I collagen-coated cover slip for 3 h. Cells were processed for immunofluorescence of FAK^Y397^ phosphorylation, stained with DAPI, and observed under a confocal microscope. Scale bar: 20 μm. **b** and **c** A7r5 cells, stably expressing TM-GFP, TM^S490, 492A^-GFP or GFP, were cultured to confluence. Following detachment, cells were resuspended and attached to type I collagen-coated dish for 3 h. Cell lysates were analyzed for FAK phosphorylation and expression. **b** shows a representative result of immunoblotting; (**c)** summarizes the quantitative results of FAK phosphorylation ratio and FAK expression normalized by α-tubulin levels expressed as mean ± SEM (*n* = 3)

We then examined whether TM affects FAK activation. Immunofluorescence of phosphorylated FAK was more intense in TM-GFP-expressing A7r5 cells than cells expressing GFP or TM^S490, 492A^-GFP and can be seen at focal adhesions of pseudopods (Fig. 5a). Furthermore, FAK phosphorylation was significantly increased in cells stably expressing TM-GFP, whereas cells expressing TM^S490, 492A^-GFP exhibited no difference from control. In contrast to FAK activation, the expression of FAK was not affected by TM expression in VSMCs (Fig. 5b and c).

### TM expression has no effect on PDGF-stimulated proliferation of VSMCs

**Fig. 6:**
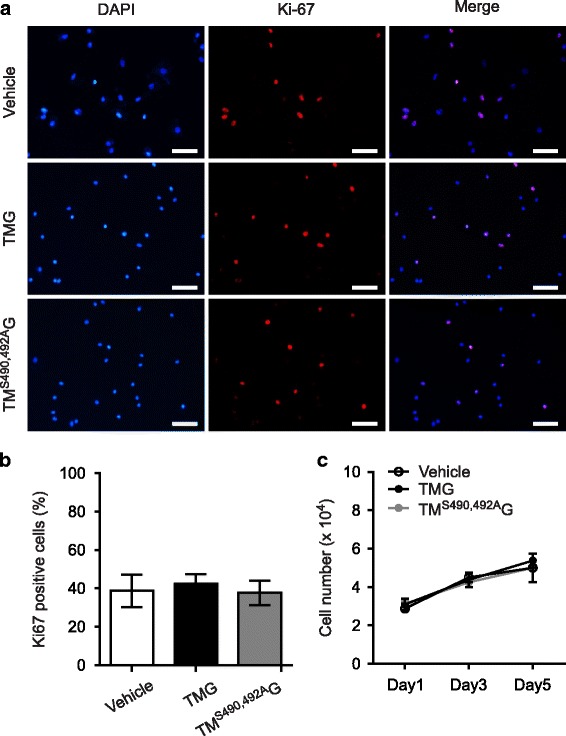
Thrombomodulin expression did not affect PDGF-stimulated proliferation of A7r5 cells. A7r5 cells were transfected with pEGFP, pEGFP-TM, or pEGFP-TM^S490,492A^, serum-starved for 48 h, and treated with PDGF-BB for 24 h. Cells were fixed and stained with DAPI (blue) and Ki-67(red). **a** shows representative results; (**b**) summarizes the ratio of Ki-67-positive cells; (**c**) summarizes total cell numbers in cells expressing vector alone, TM-GFP, and TM^S490, 492A^-GFP from at least 3 independent experiments. Results were expressed as mean ± SEM. Scale bar: 100 μm

TM was shown to decrease the proliferation of tumor cell lines derived from patients with malignant melanoma [30]. On the contrary, recombinant TM containing 6 EGF-like repeats increases DNA synthesis in ECs and VSMCs [6, 15]. To determine whether TM affects VSMCs proliferation, A7r5 cells expressing GFP-tagged TM or CS-deficient TM^S490, 492A^ were stimulated with PDGF and their entry into S phase was indicated by Ki-67 staining. The percentage of Ki-67-positive nuclei was calculated among GFP-expressing cells. In control cells, 15.8% ± 1.6% nuclei were Ki-67-positive and no difference was detected in the percentage of Ki-67-positive cells among GFP-, TM-GFP- or TM^S490, 492A^-GFP-expressing cells (Additional file 1: Supplementary data, Figure S3A and B). To substantiate these results, the percentage of Ki-67-positive cells was examined in A7r5 cells stably expressing vector only, TM-GFP, and TM^S490, 492A^-GFP. Likewise, no difference was detected in the percentage of Ki-67-positive cells (Fig. 6a and b) or cell number among three groups (Fig. 6c). These results suggested that TM expression did not affect VSMCs proliferation.

## Discussion

In this study we described a new function of TM in VSMCs. We showed that TM enhances cell adhesion, spreading, and migration of VSMCs. Moreover, we provide evidence showing that the CS moiety at the serine/threonine-rich domain of TM mediates these functions.

Smooth muscle cell migration is an integral part of vascular development, atherogenesis, and injury-induced vascular remodeling [1, 31]. Migration is a multistep process which involves cell protrusion, adhesion, spreading, and contraction [32]. During cell migration, cell adhesion to the substratum and interaction with ECM proteins play a central role in the mobile process. Extensive evidence indicates that soluble TM has roles in cell migration, thereby promoting angiogenesis and wound healing. A soluble recombinant TM fragment consisting of the second and third domains (rTMD23) stimulates ECs migration in vitro and angiogenesis both in vitro and in vivo [15]. Similarly, recombinant TM fragments consisting of the three N-terminal domains (rTMD123) and rTMD23 promote cutaneous wound healing [33]. Both cell spreading and wound closure are slower in TM-deficient keratinocytes compared to their wild-type counterpart, suggesting a role for TM in cell migration [33]. Indeed, TM was shown to mediate PDGF-stimulated migration activity of cultured bladder smooth muscle cells [9]. Furthermore, forced expression of TM promotes cell adhesion and migration of TM-deficient A2058 melanoma cells over a fibronectin-coated surface [10]. Results from this study agree fully with those detected in A2058 cells and suggest that TM enhances VSMCs motility, at least in part, through promoting cell adhesion. It’s noteworthy that TM was reported to inhibit the migration and invasion of different cancer cells [34–37], probably through its role in preventing epithelial-mesenchymal transition [35]. The mechanisms underlying these apparently opposite roles of TM in epithelial cancer cells and VSMCs warrant further investigation. Furthermore, the impact of TM-enhanced VSMCs migration activity in vascular remodeling remains to be determined as previous evidence showed that TM overexpression inhibits arterial overdilation-induced neointima formation [38].

The CS moiety bound to the Ser/Thr-rich domain is indispensable in the anti-coagulant function of TM, but whether the CS moiety of TM performs other functions remained elusive. Our results showing that removing the CS moiety, either through ChABC-catalyzed degradation or via expressing the CS-devoid mutant, eliminated the TM effect on VSMCs adhesion and migration establish a new role for the CS moiety in mediating TM-enhanced cell migration. These results are compatible with previous studies showing that CS proteoglycans facilitate the cell-cell and cell-ECM interaction during cell adhesion and migration [16–19, 39–41]. Given our previous results that the lectin-like domain of TM binds to fibronectin [10], we were surprised to find that CS deficiency, not the lectin-like domain deletion, eliminates VSMCs adhesion and migration on fibronectin (Additional file 1: Figure S2 and Fig. 3C). Furthermore, TM-enhanced cell adhesion and migration were more pronounced with fibronectin coating than collagen coating. These results implicate the CS moiety of TM in binding fibronectin, a notion supported by an earlier study showing that CS of melanoma cells binds to a specific domain of fibronectin and can either act independently or coordinate with an integrin to enhance melanoma cell adhesion to fibronectin [40].

CS proteoglycans coordinate with integrins to function in cell adhesion and migration [39–41]. Reduction of endogenous CS proteoglycan expression inhibits the motility, migration, and adhesion of fibrosarcoma cells, whereas treatment with exogenous CS proteoglycans dose-dependently stimulates cell motility and migration via a JNK-dependent pathway [18]. A recent study showed that chondroitin/dermatan sulfate proteoglycans play a key role in the adhesion and migration of VSMCs. The DS-epi1^−/−^ VSMCs, which exhibit reduced iduronic acid both on the cell surface and in secreted CS proteoglycans, have fewer focal adhesion sites, decrease directional migration, and reduce the activation and expression of FAK compared to the wild-type VSMCs [19]. The activation of FAK downstream of β_1_ integrin signaling is a major pathway underlying cell migration activity. Our results that β_1_ integrin mediates TM-enhanced VSMCs migration and the fact that TM-enhanced FAK activation is dependent on the CS moiety again point to the activation of β_1_ integrin-FAK in mediating TM-enhanced VSMCs migration. In this context, the serine/threonine-rich domain of TM was shown to interact with β_2_ integrin expressed on peripheral blood mononuclear cells in vitro [42]. Further study is required to unravel how the CS moiety of TM interacts with integrin-FAK pathway as we were unable to detect the binding complex between TM and β_1_ integrin (CP Pai and MJ Jiang, unpublished results).

The role of TM in cell proliferation appears to vary depending on the form of TM expressed and secreted by cells. A soluble TM fragment consisting of the EGF-like repeats (rTMD2) stimulates proliferation in various cell types, including ECs, VSMCs, and Swiss 3T3 cells [6, 15, 43], and was shown to act through a fibroblast growth factor receptor in ECs [44]. On the contrary, membrane-bound TM overexpression and a soluble recombinant TM fragment consisting of the entire extracellular domains inhibit both thrombin-stimulated VSMC proliferation in culture [45, 46] and vascular injury-induced neointima formation [38, 47]. These contradictory results illustrate the complex roles of TM reflecting the interaction of various domains of TM with other proteins and possibly among different TM domains. Our results are consistent with those reported in cultured urinary bladder smooth muscle cells, both showing no effect of increased TM expression on PDGF-stimulated cell proliferation [9]. It is well established that TM soluble fragments are detected in serum and urine in various forms and concentrations depending on physiological conditions [48]. Therefore, the role of TM in VSMCs proliferation in vivo may vary under different pathophysiological conditions.

## Conclusion

The CS moiety at the serine/threonine-rich domain mediates TM-enhanced VSMCs adhesion, spreading, and migration activity through the activation of β_1_ integrin and a FAK pathway.

## Additional file

Additional file 1: Supplementary data. (DOCX 315 kb)

## Abbreviations

BSA: Bovine serum albumin
ChABC: Chondroitinase ABC
CS: Chondroitin sulfate
ECM: Extracellular matrix
ECs: Endothelial cells
EGF: Epidermal growth factor
FAK: Focal adhesion kinase
GAPDH: Glyceraldehyde 3-phosphate dehydrogenase
GFP: Green fluorescence protein
HASMCs: Human aortic smooth muscle cells
PBS: Phosphate buffered saline
PDGF: Platelet-derived growth factor
shRNA: Short hairpin-type RNA interference
TM: Thrombomodulin
VSMCs: Vascular smooth muscle cells
