# Supplementary material for: The chondroitin sulfate moiety mediates thrombomodulin-enhanced adhesion and migration of vascular smooth muscle cells
Source: J Biomed Sci. 2018 Feb 13;25:14. doi: 10.1186/s12929-018-0415-7 (PMC5809974; doi:10.1186/s12929-018-0415-7)
Supplement: Supplementary file 1 — Supplementary data. (DOCX 315 kb) [file 12929_2018_415_MOESM1_ESM.docx]

**RNA extraction and semiquantitative RT-PCR**

Total RNA from A7r5 cells was isolated using TRI REAGENT. The first-strand cDNA was synthesized from 2 µg of total RNA. PCR was performed using 200 ng of cDNA with the following thermal profile: 1 min 94 ^o^C, 1 min annealing, 1 min 72 ^o^C, and a final elongation step at 72 ^o^C for 15 min. TM and GAPDH fragments were amplified with primer concentration of 400 nM and 40 nM, respectively. **PCR** products were resolved on 2% agarose gels as previously described [[1](#_ENREF_1)].

**Cell adhesion assay**

The ability of A7r5 cells to adhere to type I collagen or fibronectin was quantified according to a published method [[2](#_ENREF_2)]. Clear-bottom 96-well plates were coated with 40 µg/ml collagen type I or 5 µg/ml fibronectin in PBS for 16 h at 4 ^o^C. Non-specific binding site were blocked with 2% BSA in PBS. A7r5 cells were transfected with 1µg of pEGFP, pEGFP-TM, pEGFP-TMΔL or pEGFP-TM^S490, 492A^ for 12 h and cultured for 36 h. The detached cells (6 x 10^3^ cells/100 µl) were added to the plain or coated 96 well plates and incubated for 30 min at 37 ^o^C. Non-adherent cells were removed by washing with DMEM. The cells were fixed with 4% paraformaldehyde, stained with 0.1% crystal violet for 25 min, and solubilized with 0.5% triton X-100. The optical density at 595 nm (OD 595) was determined using a microplate reader.

References:

[1] I.C. Lo, T.M. Lin, L.H. Chou, S.L. Liu, L.W. Wu, G.Y. Shi, et al., Ets-1 mediates platelet-derived growth factor-BB-induced thrombomodulin expression in human vascular smooth muscle cells, Cardiovasc Res. 81 (2009) 771-779.

[2] K. Lessan, D.J. Aguiar, T. Oegema, L. Siebenson, A.P. Skubitz, CD44 and beta1 integrin mediate ovarian carcinoma cell adhesion to peritoneal mesothelial cells, Am J Pathol. 154 (1999) 1525-1537.

**Table S1**. Primer sequence, annealing temperature, and PCR cycles for rat TM and GAPDH.

| Primer |  | Sequence | Annealing temperature (^o^C) | PCR cycles | Product size (bp) |
| --- | --- | --- | --- | --- | --- |
| Rat TM | Sense | CCATCTTCAGACATATTGGGC | 60 | 30 | 199 |
|  | Reverse | GCCATATCTCCCTAAGCAG |  |  |  |
| Rat GAPDH | Sense | GTTCCAGTATGACTCTACCC | 58 | 25 | 424 |
|  | Reverse | ACTCTTCTGAGTGGCAGTGATGGC |  |  |  |

**
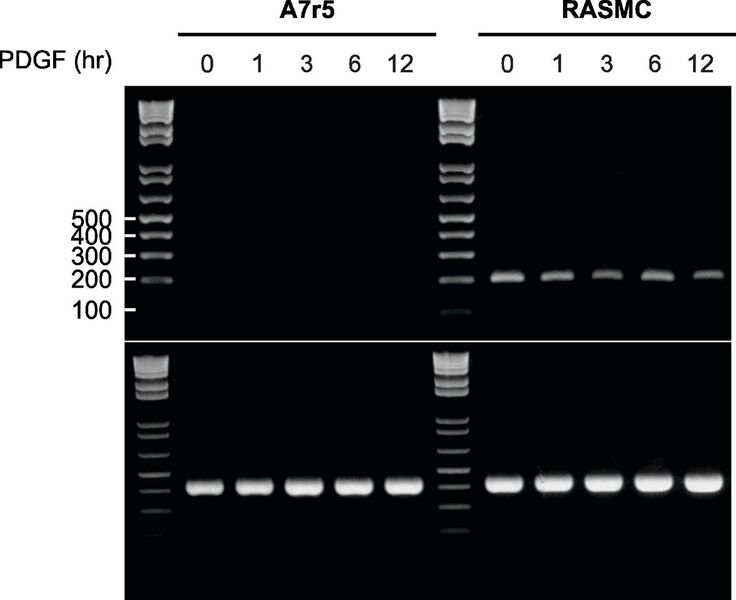
**

**Figure S1**. Representative RT-PCR analysis of TM mRNA expression in A7r5 cells and RASMCs. A7r5 and rat aortic smooth muscle cells (RASMCs) were serum-starved for 2 days and stimulated with PDGF (10 ng/ml) for 0, 1, 3, 6 and 12 h. RT-PCR products for TM with expected size of 199 bp were detected in RASMC but not in A7r5 cells. GAPDH mRNA RT-PCR products were examined as the internal control. The molecular size marker is a 1-kb DNA ladder.

**
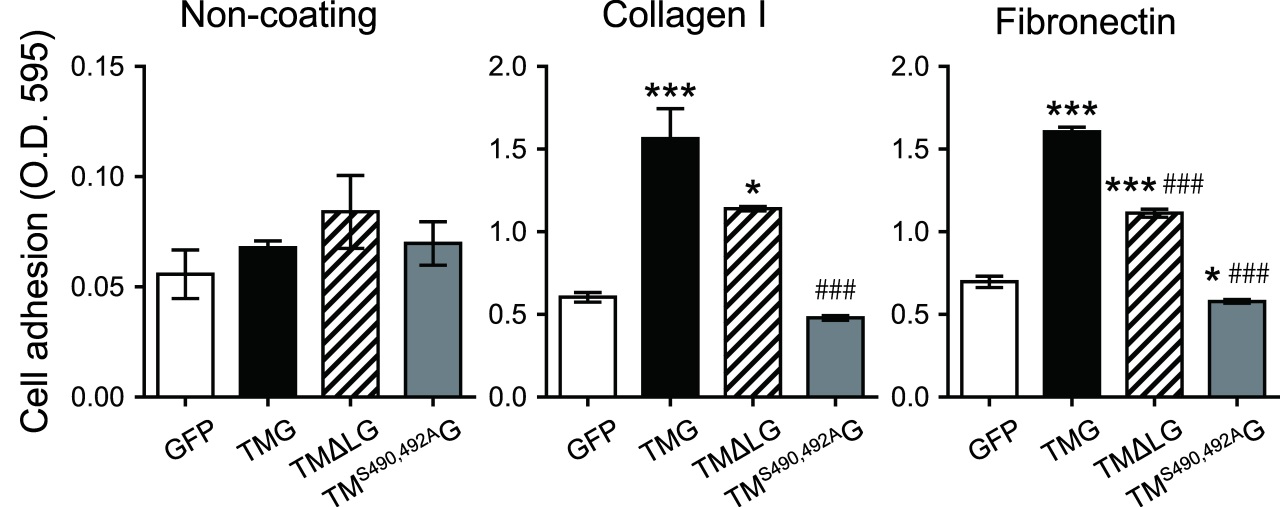
**

**Figure S2.** Thrombomodulin (TM) enhanced VSMCs adhesion in a chondroitin sulfate-dependent manner. A7r5 cells were transfected with pEGFP, pEGFP-TM, pEGFP-TMΔL or pEGFP-TM^S490, 492A^ for 12 h and cultured for 36 h. The cells (6 x 10^3^ cells) were added to wells without coating, or coated with type I collagen or fibronectin, and incubated for 30 min at 37 ^o^C. The adhered cells were fixed, stained with 0.1% crystal violet, and solubilized with 0.5% triton X-100. The optical density at 595 nm (OD 595) was determined using a microplate reader. Results were expressed as mean ± SEM (n = 3). **P*<0.05, ****P*<0.001, compared with the GFP-expressing group; ^###^*P*<0.001, compared with the TM-GFP-expressing group.

**
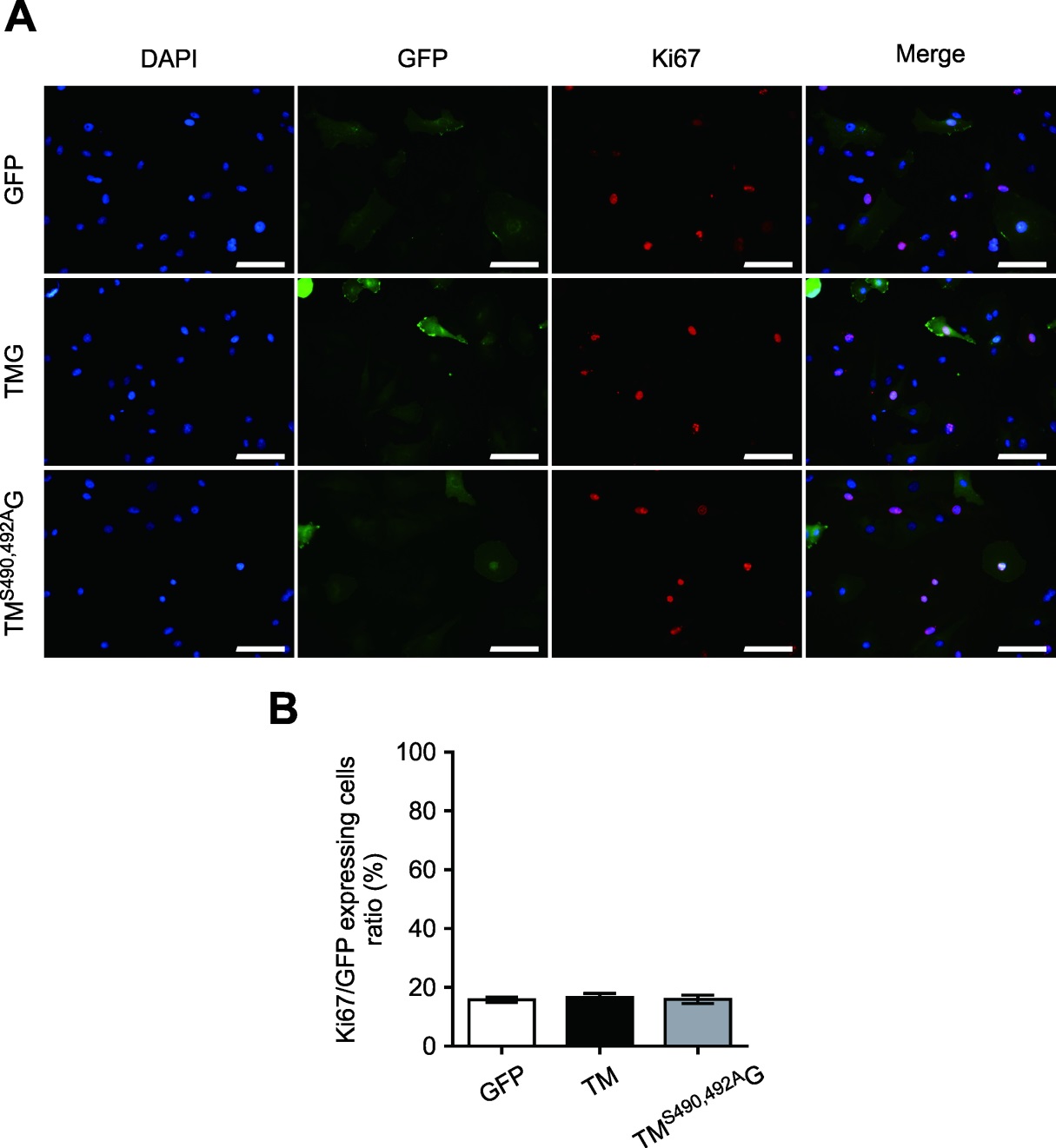
**

**Figure S3**. Thrombomodulin (TM) overexpression did not affect PDGF-stimulated proliferation of A7r5 cells. A7r5 cells were transfected with 1 µg of pEGFP, pEGFP-TM, or pEGFP-TM^S490,492A^ for 24 h, serum-starved for 48 h, and treated with 10 ng/ml PDGF-BB for 24 h. Cells were fixed with 4% paraformaldehyde and then stained with Ki-67(red) and DAPI (blue). **A** shows representative results and **B** summarizes the ratio of Ki-67-positive cells in cells expressing vector alone, TM-GFP, and TM^S490, 492A^-GFP from at least 3 independent experiments. Results were expressed as mean ± SEM. Scale bar: 100 µm.
